# Aural Manifestations of Antineutrophil Cytoplasmic Antibody (ANCA)-Associated Vasculitis—Diagnosis, Symptoms, Treatment

**DOI:** 10.3390/jcm13154298

**Published:** 2024-07-23

**Authors:** Michał S. Kaczmarczyk, Dariusz Jurkiewicz, Stanisław Niemczyk, Aleksandra Rymarz

**Affiliations:** 1 Department of Otolaryngology and Laryngological Oncology with Clinical Department of Craniofacial Surgery, Military Institute of Medicine—National Research Institute, Szaserów 128, 04-349 Warsaw, Poland; mkaczmarczyk@wim.mil.pl (M.S.K.); djurkiewicz@wim.mil.pl (D.J.); 2 Department of Internal Diseases, Nephrology and Dialysis, Military Institute of Medicine—National Research Institute, Szaserów 128, 04-349 Warsaw, Poland; sniemczyk@wim.mil.pl

**Keywords:** ANCA, aural manifestation, OMAAV, BVAS, VDI, facial palsy, hypertrophic pachymeningitis

## Abstract

Antineutrophil cytoplasmic antibody (ANCA)-associated vasculitis (AAV) is a group of vasculitis sharing a common pathophysiology, which affects small and medium blood vessels. There are three categories of AAV: granulomatosis with polyangiitis (GPA), microscopic polyangiitis (MPA), and eosinophilic granulomatosis with polyangiitis (EGPA). As a systemic disease, AAV can affect basically every organ. The goal of this publication is to sum up and underline the problem of the aural manifestation of AAV; it details the definition of Otitis Media with Antineutrophil Cytoplasmic Antibody Associated Vasculitis (OMAAV) and allows for a better understanding of the specific tasks of medical professionals taking part in the diagnostic and therapeutic process. Among others, this publication is directed to otolaryngologists who may encounter patients with AAV and often are the first specialists who see patients with early symptoms of AAV. This publication presents brief characteristics of AAV, descriptions of aural manifestations and symptoms, differential diagnosis, and both pharmacological and surgical treatment options, based on current recommendations and information found in the literature and clinical databases.

## 1. Characteristics of Antineutrophil Cytoplasmic Antibody-Associated Vasculitis

Antineutrophil cytoplasmic antibody (ANCA)-associated vasculitis (AAV) consists of granulomatosis with polyangiitis (GPA), microscopic polyangiitis (MPA), and eosinophilic granulomatosis with polyangiitis (EGPA). This subdivision is based on the different serology statuses and clinical characteristics of each entity [1,2,3]. Although AAV is mostly in the field of interest of rheumatologists or nephrologists, the manifestations of these units can affect every system of the human body. Sometimes, the course of AAV can be very deceptive, which can lead to a troublesome diagnosis and delayed treatment. As a consequence, irreversible organ damage or even death can occur. Consequently, it is important to remember AAV while making a diagnosis. Some signs and symptoms in the head and neck may originate from vasculitis. Otolaryngologists can encounter them performing examinations on a daily basis. Apart from granulomas seen in endoscopic or radiologic exams, as a common manifestation of AAV, one can see nasal symptoms such as septal perforations, chronic nasal bleeding, and nasal blockage due to polyps. There are also pharyngo-laryngo-tracheal symptoms such as Eustachian tube narrowing and subglottic stenosis. Last but not least, there are temporal bone manifestations. The prevalence of head and neck involvement in AAV patients differs in the literature. Depending on the type of AAV and the point in the course of the disease, the involvement of the head and neck can vary from approximately 80 to 90% in granulomatosis with polyangiitis [4,5,6,7,8,9,10,11,12,13], from 50 to 90% in eosinophilic granulomatosis with polyangiitis [10,14,15,16,17,18,19], and from 20 to 50% in microscopic polyangiitis [11,17,20,21]. Research shows that there is no adequate biological marker of vasculitides, and the most important factor in diagnosing and activity checking is the clinical evaluation of the patient. Diagnosing ANCA vasculitis requires cooperation between internal medicine doctors and other specialists. This allows for knowing the signs and symptoms that are sought in every organ system. So, to do this, the special scoring systems listed below have been proposed, and familiarizing oneself with them seems to be necessary. Both the American College of Rheumatology/European League Against Rheumatism (ACR/EULAR) ANCA criteria and diagnostic tools like the Birmingham Vasculitis Activity Score (BVAS) (including its alterations for granulomatosis with polyangiitis or pediatric vasculitides) or the vasculitis damage index (VDI) are commonly used in internal medicine to either diagnose or evaluate the activity of the disease. They consider clinical symptoms from every system of the human body. The ACR/EULAR criteria are used to make a diagnosis [22]. The BVAS scale is used to describe current and recent disease activity and the VDI focuses on persistent organ damage [23,24]. In the otolaryngology system, the BVAS scale concentrates on bloody nasal discharge/crusts/ulcers/granulomata, paranasal sinus involvement (including radiological imaging), subglottic stenosis with stridor or hoarseness, conductive hearing loss, and/or sensorineural hearing loss, with the last one highlighted as a major symptom. Upon the first evaluation of vasculitis, all features due to active vasculitis are scored irrespective of how long they have been present. Later evaluations take into account only those symptoms that are new or have become worse in the preceding four weeks. The “none” box is reserved for patients without any signs and symptoms in a particular organ system. In version 3 of BVAS, the definition of “persistent disease” was added. The “persistent” box at the end of the questionnaire can be ticked only if there are no new/worse items presented in the whole form. Even one new feature in one of the ten general sections excludes the possibility of marking this box. Signs and symptoms should be listed as persistent disease if they last up to three months. It is very important to differentiate between active and persistent disease, as the first one needs an escalation of the therapy and the second state does not require increasing immunosuppression but only maintaining it. A mixture of new/worse and persistent symptoms has to be treated as an active disease. Moreover, persistent disease has to be distinguished from irreversible damage. Items present longer than three months are considered as constituting damage and should be scored separately in the vasculitis damage index (VDI). Firstly, VDI can be assessed 3 months after the diagnosis of ANCA vasculitis and it describes organ dysfunction irrespective of a connection with the disease. VDI otolaryngologic signs and symptoms seem to overlap with those in BVAS: hearing loss (conductive or sensorineural), nasal blockage/chronic discharge/crusting, nasal bridge collapse/septal perforation, chronic sinusitis (including radiological changes), and subglottal stenosis with or without surgery. As time passes by, the VDI value can only remain stable or deteriorate. Although it is important to mention that not every symptom persisting for more than 3 months is acquired as damage, nasal bleeding or crust, for example, cannot be treated that way. In this case, it is a persistent activity of the underlying disease. In this article, we would like to focus on aural symptoms, their diagnosis, and treatment.

## 2. Aural Manifestations of ANCA Vasculitis

Aural signs are seen in GPA in about 30–60% of cases [6,7,9,11,12,20,25], in MPA in 10–30% of cases [11,20,26], and in EGPA in 10–40% of cases [19,27] during the course of the disease. Moreover, otologic symptoms can appear as an initial symptom in 40 to 80% of AAV cases [28]. Many years have passed since the establishment of the requirements for ANCA-related vasculitis disease in the ears. In 2014, the concept of Otitis Media with Antineutrophil Cytoplasmic Antibody Associated Vasculitis (OMAAV) was proposed [25,29,30]. In 2016, the Japan Otological Society established diagnostic criteria for this new entity based on a survey conducted in Japanese society between 2013 and 2014 [29,30]. OMAAV is diagnosed if at least one point from categories A and B is fulfilled and there is a lack of features from category C.

### OMAAV Diagnostic Criteria

All three criteria (A, B, and C) must be fulfilled:

(A) At least one of the following clinical courses is noted:

1. Intractable otitis media with effusion or granulation, which is resistant to antibiotics and the insertion of a tympanostomy tube.
2. The progressive deterioration of bone conduction hearing levels.

(B) At least one of the following features is true:

1. Already diagnosed as AAV (GPA, MPA, or EGPA) based on the involvement of other organs.
2. Positivity for serum MPO- or PR3-ANCA.
3. Histopathology consistent with AAV, i.e., necrotizing vasculitis predominantly affecting small vessels with or without granulomatous extravascular inflammation.
4. At least one of the following accompanying signs/symptoms of AAV-related involvement is witnessed:
  - Involvement with upper airway tracts other than ears, scleritis, lungs, and/or kidneys;
  - Facial palsy;
  - Hypertrophic pachymeningitis;
  - Multiple mononeuropathy;
  - Transient alleviation of symptoms/signs with administration of 0.5–1.0 mg/kg prednisolone and relapse with discontinuation of treatment.

(C) Differential diagnosis

1. Cholesteatoma;
2. Cholesterol granuloma;
3. Eosinophilic otitis media;
4. Tuberculosis;
5. Malignant otitis externa, skull-base osteomyelitis;
6. Neoplasms (malignancy, inflammatory myofibroblastic tumor, etc.);
7. Otitis media or inner ear inflammation caused by autoimmune diseases and vasculitis other than AAV.

OMAAV is divided into local and systemic forms. The first one is limited to the ear and it usually starts with the ear, but it can precede systemic vasculitis. It is perceived as a deceptive condition because of its rarity and lack of specificity, which make up for its diagnostic hardship. Consequently, the local form is often first seen by otolaryngologists. The second form seems to be easier to diagnose as it starts as a systemic reaction that also occupies the temporal bone, but it cannot be underestimated by investigators. 

Aural manifestations of ANCA vasculitis are as follows: otitis media with effusion, chronic otitis media, sensorineural hearing loss, facial palsy, chronic or acute vestibular disorders, and different forms of external otitis [7,12,31]. Recently, cases of hypertrophic pachymeningitis are regarded as temporal symptoms of OMAAV [32].

Otitis media with effusion (OME) is one of the most common entities diagnosed by otolaryngologists worldwide. What is more, it is the most frequent aural manifestation of AAV, often resulting in hearing loss. It usually presents as a presence of serous or mucous liquid behind the eardrum often associated with underpressure in the tympanic cavity. Most cases are asymptomatic, but it can also manifest as otalgia, a feeling of fullness in the ear, or mild hearing conductive loss. As for being harmless, OME is usually taken under surveillance for about 3–6 months after excluding a nasopharynx lesion and short-course treatment for Eustachian tube inflammation [33]. After performing an otological examination and tympanometry with an audiogram, further treatment is proposed. It mostly comes down to inserting a tympanostomy tube. Something good to know is that OME can also be a symptom of OMAAV. This modest aural abnormality, when idiopathic and often asymptomatic, can be problematic, relentless, and hard to treat in the case of an OMAAV cause. What is more, it also can exacerbate into more acute inflammation or prolong into chronic otitis media with granulation, with all its complications. It is said that OME is encountered in 49% of patients with OMAAV [29,34]. Research underlines that repetitive OME can be a serious diagnostic problem. That is why solutions such as the scoring system of OMAAV tympanic membrane (SCOT) were proposed to evaluate tympanic membrane and external acoustic meatus findings and to differentiate one from another [34]. This system is based on the evaluation of the thickening of pars tensa, vasodilation of pars tensa, and posterior wall swelling. Then, each of the factors is marked from 0 to 3, where 3 stands for undetermined evaluation due to posterior wall swelling. The cut-off value of the total score set at 2 points had the best combination of sensitivity (93.0%) and specificity (74.0%) to distinguish OMAAV from OME. Although, the thickening of pars tensa, vasodilation of pars tensa, and posterior wall swelling might also be seen in other otologic diseases such as cholesteatoma, tuberculosis, cholesterol granuloma, and eosinophilic otitis media. The SCOT scoring system can be handy in differentiating OMAAV from repetitive OME in adults. Another handy diagnostic tool is the measurement of the ETosis-derived products in the otitis media fluid. As there is an absence of ETosis-derived products in patients with OME caused by the dysfunction of the eustachian tube, this utility can differentiate OMAAV from OME. There was also a strong correlation between the MPO-DNA complex in the middle ear fluid titer and the air and bone conduction thresholds. On the contrary, serum levels of ANCA and inflammation indicators have not shown that connection [35].

Chronic otitis media (COM) with granulation can be another otological manifestation of OMAAV. The definition of COM is the preservation of tympanic membrane perforation for more than 3 months. An association with granulation tissue is a sign of irreversible and active inflammation. Granulation tissue may block the airflow drainage of the middle ear and after inflammation exacerbations, causing bone erosion. The reasons for chronic otitis media are unknown, but it is clear that chronic inflammation, as it occurs in AAV, is a fundamental factor. It is said that chronic otitis media with granulation can be found in 44% of patients with OMAAV [29]. Although the time to diagnose idiopathic chronic otitis media is by definition 3 months, it is crucial to start the diagnostic evaluation of ANCA vasculitis sooner, especially with active inflammation, granulation tissue, and sensorineural hearing loss not responding to standard treatment.

Hearing loss in OMAAV comprises conductive hearing loss, sensorineural hearing loss (SNHL) including complete deafness, and mixed hearing loss. It is the most common aural symptom encountered in AAV [4,6,11,29]. It is known that conductive hearing loss is caused by edema, fluid, pathologic tissue masses, or auditory ossicle deviations in the tympanic cavity. Some cases can be diagnosed with otoscopy and radiology. As mentioned before, OME and COM are the main causes of conductive hearing loss. On the other hand, the direct trigger of SNHL is not clear, but is it known that the ear condition progresses after myringotomy, mastoidectomy, or infections [25]. The outcomes of the causal treatment of hearing loss in OMAAV, based on a 2014 Japanese nationwide survey with almost 300 patients, are not yet satisfactory, because about 40% of patients do not respond to treatment at all and another 30% respond to treatment only partially. What is more, complete deafness has the worst prognosis of them all [29]. Sensorineural hearing loss is an important problem for audiology and otolaryngology. Either sudden or slowly, increasing hearing loss is always dreadful for patients as it can seriously handicap their daily functioning. This diagnosis is made through audiometry, where bone conduction thresholds are elevated. Generally, the most common SNHL cause is Sudden Sensorineural Hearing Loss (SSNHL). Usually, it is regarded as idiopathic and treated with steroids or hyperbaric therapy. In the case of misdiagnosis with SNHL associated with OMAAV, the effect of steroid treatment is short-lived or incomplete and also reduces the ANCA titer [25]. However, there are some signs that drive our diagnosis from idiopathic to specific-cause. Bilaterality, insensitivity to common treatment, or additional symptoms can lead us towards other diagnoses—for example, OMAAV. Slowly, progressive sensorineural hearing loss can indicate the destruction of the inner ear or the distal segment of the VIII nerve. In SNHL, the studies have shown that the inflammatory process includes the cochlea, vestibule, and facial nerve, due to their proximity to the medial ear. The inflammation usually progresses to the inner ear through its natural vulnerabilities such as oval or round windows. When it comes to sensorineural hearing loss, it seems that hemorrhage within the cochlea (mostly spiral ligament) and capillary occlusion with thickened walls in the stria vascularis and spiral ligament are the main causes [36]. The mechanism of cochlear hearing loss was proposed, which was based on the infringement of K+ ion homeostasis usually maintained via stria vascularis and associated tissues. Firstly, it is a reversible state, which can progress to the irreversible state of the dysfunction of the capillaries and the permanent damage of hair cells [25,37]. There is also a possibility of finding some neuronal damage in the VIII nerve, especially among patients with hypertrophic pachymeningitis [7,12,13,27,30,38]. However, histopathological findings have not confirmed vestibulocochlear nerve involvement via inflammation [36]. Radiologic findings seem to agree with histopathology. FLAIR MRI and MRI (CE) contrast enhancement are used to evaluate inner ear disturbances. The signals from the cochlea are stronger on the side with the worse hearing level/stronger symptoms, due to blood–labyrinthine/nerve barrier damage [37,39,40]. There is also a case of using PET/FDG-CT to detect inflammatory processes in the middle ear, which is not clear using MRI CE. In correlation with ANCA and other symptoms, it is proven to distinguish OMAAV from noninflammatory disturbances [41].

Another important sign of OMAAV can be facial palsy (FP). Like other cranial nerves, the facial nerve could be involved in ANCA vasculitis [12,13,19,31,32,38,42]. The prevalence of cranial nerve involvement is lower than peripheral nerve involvement, and it is generally estimated to be approximately 5–10% [10,13,14,19,38]; it could be higher in patients with OMAAV. Facial palsy with hypertrophic pachymeningitis is not only a part of the diagnostic criteria, regarded as equally important to ANCA serology or histopathology, but is also a disturbing sign for the patient and for the clinician. Putting aside life-threatening central facial nerve palsy, peripheral facial nerve palsy also has to be properly diagnosed due to its possible irreversibility, neoplastic cause, or local complications. The Japanese national survey has shown that facial palsy was presented as an initial symptom in 18% and in 32% during clinical observation [29,43]. Unlike Bell’s Palsy, which is considered idiopathic, facial palsy in OMAAV is often recurrent, sometimes bilateral [43] and refractory to standard treatment. Keishi Fujiwara et al. have found that facial palsy is more frequent among women and is related to the occurrence of hypertrophic pachymeningitis [44]. Taro Fujikawa et al. used CE-3D-GRE MRI to assess the activity of vasculitis in the middle ear by evaluating the enhancement of the fallopian canal in tympanic and mastoid parts. Although it was not related to facial palsy, it gave promising results [39]. However, the mechanism of facial paresis in OMAAV is still unknown, there are three main assumptions that partially find confirmation in histopathology. First, the granulation of the fallopian canal is ipsilateral to the affected side. Second is the vasculitis of the vasa nervorum and last is the occurrence of hypertrophic pachymeningitis apart from the occupied side [30]. During the histopathologic exam, vasculitis was seen in the vasa nervorum of the tympanic and mastoid facial nerve segments with and without the clinical correlation of facial paresis. There was also the vacuolization of the vertical segment of the facial nerve [36,44]. In cases of masked mastoiditis with FP and those without improvement or recurrences after low steroid doses and surgery, ANCA titers should be checked [45].

Vertigo and dizziness are encountered by about 30% of patients with OMAAV [29,46]. The cause of vertigo is due to the involvement of the vestibular portion of the inner ear. It is said that it can affect about 90% of patients with OMAAV, apart from being symptomatic or not [46]. On the other hand, dizziness results from central nervous system infiltration [47]. The symptoms can present as both acute vestibular syndrome or the chronic and progressive deterioration of vestibular organs, and dizziness. During histological examination, hemorrhage was seen in the stroma of the cristae of semicircular canals. Also, hemorrhage and inflammatory cells were identified in the vestibule. Some kind of degeneration of the cupulae, destruction of ampullae, or loss of dendrites was described. Authors have suggested that inflammation is related to acute hemorrhage rather than neuropathy, unlike the cochlear one [36]. When it comes to diagnostics, the most common form is caloric testing. Other tests are also in use, for instance, the eye tracking test, vestibulo-ocular reflex test, and optokinetic nystagmus test [46]. Recently, vHIT was proposed as the most appropriate test for semicircular canal function evaluation for patients with OMAAV, especially those with OME or COM with granulation. This is due to the deterioration in the thermal transmission of the stimulus through the fluid or inflamed tissue. Outcomes of vHIT use show that there is significantly higher involvement in the posterior semicircular canals than in the other two canals. The authors claim this results from differences in the blood supply of semicircular canals [48].

Hypertrophic pachymeningitis (HP) is a thickening of dura matter in the case of its chronic inflammation. As an entity, it can be isolated, sometimes called idiopathic, or it might be secondary to other diseases. HP has an undetermined pathogenesis, but it is known that it was linked with the following conditions: systemic inflammatory diseases like ANCA vasculitides, Sjogren’s syndrome, rheumatoid arthritis, and IgG4-related disease [32,42,49]. In a nationwide survey of hypertrophic pachymeningitis in Japan, the most common cause is ANCA-related HP, making up as much as 34.0% of all cases, and the second most common cause is IgG4/ MFS-related HP [50]. The thickening of dura matter as a reactive reaction to the neoplastic process or compensation for the decreased volume of the CSF space in intracranial hypotension must be distinguished from a potentially dangerous state of hypertrophic pachymeningitis [42,49]. In OMAAV, hypertrophic pachymeningitis is seen as an initial condition in 15% of cases, and in 24% to 47 overall [29,32]. HP can be asymptomatic or it can proceed with headaches (35.2% as an initial symptom to 71,1% during the entire course); ophthalmological symptoms, such as visual loss and double vision (as an initial symptom in 13.2% and 12.6%, respectively, to one-third of HP patients during the entire course); or cranial nerve involvement (62.3%), including the vestibulocochlear nerve (9.4–27.3%) and fever in one-quarter of HP patients [29,50]. Rarely, it can lead to seizure, ataxia, and sensory loss [42]. This diagnosis is established using dura matter biopsy or the thickening and enhancement of the dura mater on T1-weighted gadolinium-enhanced MRI. Moreover, it seems that the location, distribution, and pattern of enhancement are not random, and they are strongly related to the cause of HP [32,42]. In the case of OMAAV, there has to be a relation between the severity of local inflammation in the middle ear and adjacent intracranial areas. Morita et al. emphasize the importance of lesions in the internal auditory meatus, which may be a characteristic finding in OMAAV [32]. Other, OMAAV-related areas include the middle and posterior cranial fossa. As it was mentioned before, biopsies and MRI CE are used in the diagnosis of HP, which seem not to be cost-effective and convenient means of diagnostic methods. So, new strategies are being developed to extract groups of patients who are meant to have HP and widen screening towards it. These include being symptomatic, but only headaches were statistically important (rarely presented in the acute phase of otitis media [29]), and the NLR, which stands for the Neutrophil-to-Lymphocyte Ratio, and it is suggested that an NLR greater than 3.46 is highly specific for HP [32]. Moreover, research shows that the higher occurrence of otitis media with otorrhea and sinusitis and the lower involvement of the lungs were significant risk factors for predicting the onset of HP [30,32,49]. ANCA negativity and facial nerve palsy are also associated with hypertrophic pachymeningitis [29,30]. The utility of systemic inflammatory responses is rather unclear [32,50].

## 3. Diagnosis

In the nationwide survey conducted by the Japan Otological Society between December 2013 and February 2014 consisting of 297 patients with OMAAV, the researchers recorded the most common initial symptoms, which include hearing loss 295/297 (99%), otorrhea 135/297 (45%), otalgia 99/291 (34%), tinnitus 140/282 (50%), vertigo or dizziness 74/295 (25%), and headache 70/224 (24%) [29,30]. In another study with 123 patients, the primary aural symptoms were hearing loss (75%), otalgia (12%), otorrhea (9%), aural fullness (6%), peripheral facial palsy (5%), headache (3%), tinnitus (2%), and nasal discharge [25].

The symptoms mentioned above are not specific to any condition, so a comparison with other patient’s data is required. When it comes to serology, the most frequent symptom is the predominance of MPO-ANCA positivity (56%), followed by PR3-ANCA positivity (23%), negativity for both MPO-ANCA and PR3-ANCA (17%), and positivity for both MPO-ANCA and PR3-ANCA (5%) [29,30]. Similar to GPA, EGPA and MPA ANCA titers are often not very high in the first episode of OMAAV of the middle ear, and small doses of glucocorticoids result in negative ANCA titers [25,51]. Also, double-negative ANCA can result from the low sensitivity of current methods or the yet undiscovered specificity of ANCA antibodies such as BPI (bactericidal/permeability-increasing protein)-ANCA positivity and elastase–ANCA positivity [30]. In ANCA-negative cases, ANCA should also be tested with a different technique such as new-generation ELISA and IIF [52]. Despite finding serologically double-negative patients and negative biopsy results, authors emphasize the importance of other accompanying signs/symptoms of AAV-related involvement or even the transient alleviation of symptoms/signs with the administration of 0.5–1.0 mg/kg prednisolone and relapse with the discontinuation of treatment as significant requirements of diagnosis equally important as others mentioned in point “B” [29]. Consequently, it is needed to repeatedly check ANCA titers and in some cases take a biopsy.

Upper respiratory tract biopsy specimens have low sensitivity and specificity, becoming positive in up to one-third of cases [30]. The lowest sensitivity is described in temporal bone specimens. However, it could clearly rule out other potential diagnoses [45].

Moreover, patients should also be addressed by an internal medicine doctor, who periodically checks other possible manifestations of ANCA vasculitides and evaluates them with previously mentioned diagnostic tools like BVAS and VDI.

### Temporal Bone Radiologic Findings

To summarize the information written down in the previous section, radiologic findings should always correlate with signs and symptoms. There are no pathognomonic signs in computed tomography and magnetic resonance imaging in OMAAV. The radiologic modalities that are usually used in diagnosing ANCA vasculitides in the head and neck are computer tomography without contrast and magnetic resonance imaging either with or without contrast enhancement.

Computed tomography of the head without contrast enhancement

- Hearing loss/ear fullness with the opacification of temporal air cells, without bone destruction and an intact ossicular chain, implies OME (either an acute or chronic phase).
- Pain of the mastoid, purulent and/or bloody discharge, acute hearing loss, fever, and elevated inflammation markers, with the opacification of temporal air cells, without bone destruction, and an intact ossicular chain suggest acute otitis media.
- Purulent and/or bloody discharge, typically of an unpleasant smell, usually without or with moderate pain, chronic hearing loss without fever, and signs of acute inflammation with the opacification of temporal air cells, with bone and ossicular chain destruction, indicate chronic otitis media with or without cholesteatoma.
- Chronic vertigo and sometimes concomitant hearing loss, usually without spontaneous nystagmus and sympathetic symptoms, suggest chronic labyrinthitis in the final stage of the ossification of the inner ear.

Magnetic resonance imaging of the head with or without contrast enhancement. It is mentioned when there is a need for contrast enhancement.

- Hearing loss/ear fullness with opacification in computer tomography and fluid signal in the middle ear cavity and mastoid antrum in magnetic resonance suggest OME or acute otitis media when there are additional symptoms like the pain of the mastoid, purulent and/or bloody discharge, acute hearing loss, fever, and elevated inflammation markers or intra-/extratemporal complications of otitis media. Although magnetic resonance is not required for the diagnosis of OME and acute otitis media, it should be performed when complications are suspected.
- In patients with chronic otitis media, magnetic resonance is not usually needed. It could be helpful in cholesteatoma/neoplasm differentiation or if intra-/extratemporal complications are suspected.
  - In cholesteatoma, non-echoplanar diffusion-weighted imaging (non-EP DWI, with a b parameter of 800–1000) with Apparent Diffusion Coefficient (ADC) map modalities are helpful. Lesions that are hyperintensive in T2 and non-EP DWI but hypointensive in ADC are most likely cholesteatomas.
  - Acute labyrinthitis with sudden symptoms of vertigo with nausea/vomiting, hearing loss, and nystagmus is seen as an enhancement of the inflamed inner ear in MRI with contrast enhancement.
  - Chronic labyrinthitis with the symptoms listed before. Whereas the bony obliteration of the inner ear is readily identified using CT, fibrous obliteration is recognizable only using MR imaging. In the T2 images, the high signal seen within the normal inner ear structures is absent, therefore making the involved structures no longer recognizable.
  - In peripheral facial nerve paresis, magnetic resonance is the modality of choice in the case of facial nerve paresis. CT can only indirectly assess the intratemporal part of the facial nerve; magnetic resonance can assess all segments of the VII cranial nerve. Contrast enhancement and/or a specific type of MR imaging are used depending on the localization of the facial nerve segment. Three-dimensional heavily T2-weighted MR sequences are usually used to describe the facial nerve in its cisternal and intracanalicular segments. When it is healthy, it is usually depicted as a dark linear structure against bright cerebrospinal fluid. The thickening of the nerve into the internal auditory canal and the inner ear on the affected side should be treated as pathology. On post-contrast T1-weighted images, the facial nerve is the only cranial nerve that may show normal post-contrast enhancement, sometimes asymmetric, due to arteriovenous plexus around certain parts of the nerve. Typical sites of enhancement in healthy patients are the fundal canalicular, anterior genu, and posterior genu with the proximal mastoid segment. Meatal, intralabyrinthic, and extracranial parts should show no enhancement in healthy individuals. In cases of idiopathic paresis or viral neuritis, the postgadolinum enhancement is seen from the distal internal auditory canal (intralabyrinthine segment) to the distal tympanic segment and it is called “fundal tuft sign”. The asymmetric enhancement of the geniculate ganglion can also be observed on the affected site. Although there is a pathological enhancement in contrast-enhanced 3D MRI with gradient-echo sequences (CE-3D-GRE), in ANCA patients with OMAAV syndrome, particularly in tympanic and mastoid segments, which corresponds to the intensity of middle ear inflammation, it is not connected with facial nerve paresis [39]. On the other hand, Kato et al. used 3D-FLAIR MRI to assess patients with facial nerve paresis and the conclusion was that the post-contrast enhancement was connected with facial nerve inflammation and paresis [53]. More studies in this field should be conducted to establish clear guidelines. Moreover, facial nerve paresis can be one of the symptoms of hypertrophic pachymeningitis—see below.
  - Hypertrophic pachymeningitis—asymptomatic or associated with headaches ophthalmological symptoms, cranial nerve involvement (including the VII and VIII cranial nerves), and fever. As it was written before, the thickening and enhancement of the dura mater on T1-weighted gadolinium-enhanced MRI suggests HP [54,55].
- Other modalities
  - PET/FDG-CT—it is helpful in clarifying the doubtful diagnosis of hypertrophic pachymeningitis in unclear MRI CE cases. It was shown as the FDG uptake in the area of the HP lesions [41].
  - ^67^Ga Scintigraphy used to diagnose hypertrophic pachymeningitis as an accumulation of isotope in dura mater [56].

## 4. GPA vs. EGPA vs. MPA—Do They Differ in Aural Manifestations?

The outcome of a nationwide Japanese study underlines that there are no differences in any clinical features among the cases classified as GPA, MPA, and EGPA. However, the discrimination among the three subgroups of AAV is important because systemic immunosuppressive treatment differs between GPA/MPA and eGPA. There are subtle differences in the serology and clinical course of AAV. For instance, the sensitivity of the titers of PR3- and MPO-ANCA is different in GPA, MPA, and EGPA. For GPA, PR3-ANCA has 66% sensitivity and MPO-ANCA has 24% sensitivity; for MPA, PR3-ANCA has 26% sensitivity and MPO-ANCA has 50–80% sensitivity; and for EGPA, PR3-ANCA has 2–3% sensitivity and MPO- ANCA has 30–40% sensitivity [25].

The predominance of MPO-ANCA tends to make EGPA more similar to MPA; on the other hand, the predominance of PR3-ANCA makes it similar to GPA. The incidence of facial nerve palsy and hypertrophic pachymeningitis is not linked with antibody type [25,44]. On the other hand, new-onset HP is seen more often in GPA patients than MPA [49]. MPO-ANCA shows predominantly OME-type otitis media and a higher incidence of otitis media [57]. Due to eosinophilic infiltration, the mucous and glue secretion in EGPA OME differs from GPA and MPA and shares some similarities with EOM [25,27,34].

According to a systematic review of EGPA, its most common accompanying manifestation is sinonasal disease [27]. This contrasts with the literature that states asthma and pulmonary disease are most often [58]. Peripheral and cranial neuropathies linked with EGPA are also accessory symptoms, which can contribute to hearing level, vestibular signs, and facial nerve function [27].

## 5. Other OMAAV

### 5.1. Anti-Thyroid Drug-Associated OMAAV

There have been about 100 cases of anti-thyroid drug (ATD) AAV. The incidence of AAV is higher among patients treated with propylthiouracil (PTU) than other ATDs [59]. AAV can be revealed after many years of pharmacological treatment and a specific trigger is unknown [60]. If AAV involves multiple organs, then it is possible that otitis media might not be reported and, thus, could be underestimated. Among patients with Graves’ disease who receive PTU, MPO-ANCA positivity has been reported to be more relevant than PR3-ANCA. When it comes to pathogenesis, it is said that there might be some similarities between MPO-ANCA and Thyreoperoxidase [61], or that the oxidation/sulfonation of the PTU molecule can activate autoimmune processes [62]. Consequently, PTU contributes to the pathogenesis of AAV, but by using other biological paths than idiopathic AAV [60]. Therefore, ATD-induced OMAAV is a rare condition. So far, there have been eight cases of ATD-induced OMAAV, seven of which were using PTU and one with carbimazole. In total, 25% of cases were unilateral and there were neither hypertrophic pachymeningitis nor fatal cases described. As opposed to traditional OMAAV, it is refractory to the standard treatment until cutting off ATD treatment. In mild cases, treatment starts from the discontinuation of ATD treatment, but with more severe or refractory symptoms, the induction of steroids and/or immunosuppressants is needed. The thyroid treatment is then usually changed to surgery or radioactive iodine. The majority of cases become ANCA-negative several months after treatment, but long-term follow-up is indicated.

### 5.2. OMAAV COVID

Similar to drugs, infections and vaccines can also induce AAV. There have been several cases of AAV following COVID-19. Unlike ATD OMAAV, there was not any predilection to either MPO or PR3 ANCA. In OMAAV cases after vaccines and infections, caution has to be preserved in terms of immunosuppressants, as they can awaken latent microbes and exacerbate infection. Usually, therapy is induced right after recovery. In the case report of OMAAV after COVID-19 vaccination (Pfizer-BioNTech), in a Japanese patient, clinicians used systemic steroids with cyclophosphamide instead of rituximab because of the concerns mentioned above. The second administration of a vaccine should be carefully considered in cases like that [63].

## 6. Differential Diagnosis

All intractable forms of otitis media (listed below) should be differentiated with OMAAV. The most important and easy to overlook are described.

- Cholesterol granuloma;
- Middle ear tumor;
- Bacterial otitis media (diabetes mellitus, old aged, *P. aeruginosa*: skull base osteomyelitis);
- Tuberculosis otitis media;
- Eosinophilic otitis media (EOM);
- IgG4-RD otitis media.

Cholesterol granuloma and middle ear tumors can be excluded through radiological and histopathological testing.

Bacterial otitis media in immunocompromised patients can mimic the course of OMAAV. However, there is generally a response to antibacterial treatment; if not, tuberculosis tests including histopathology should also be taken.

### 6.1. EOM vs. OMAAV

It is important to distinguish between these diseases because their treatment and prognosis differ. EOM is an abbreviation of eosinophilic otitis media; it is an entity linked with bronchial asthma, chronic rhinosinusitis with nasal polyps, and intractable otitis media. It is an allergic-type disease. The diagnostic criteria for EOM have been established (Section 6.1.1). As the disease progresses, signs of EOM advance into chronic otitis media with granulation. There are three otoscopy stages of the disease (named G1, G2, and G3) which correlate with hearing loss progression from conductive, to mixed, to sensorineural. The treatment—apart from controlling bronchial and nasal manifestations, bacterial superinfections, and obesity—includes systemic or preferably local steroid administration, biological treatment with (omalizumab), anti-IL-5 antibody (mepolizumab), an antibody targeting the IL-5 receptor (benralizumab), and a recombinant human antibody binding to the IL-4 receptor (dupilumab). However, the first choice of treatment is intratympanic glucocorticoids served through a tympanic puncture or ventilation tube. Sometimes myringoplasty or tympanoplasty are recommended. As mentioned before, a new possibility to diagnose OMAAV by measuring the MPO-DNA complex as NETs in middle ear effusions was recently introduced. This refers to all types of ANCA vasculitis but especially EGPA. Both EOM and EGPA patients present with accompanying asthma, chronic sinusitis, and peripheral blood and tissue eosinophilia, but they also differ in the case of treatment. That is why it is important to differentiate one from the other [51,64,65]. Although using MPO-ANCA serum positivity is the easiest way of diagnosing EGPA, there are plenty of ANCA-negative cases. Thus, the measurement of the MPO-DNA complex in middle ear fluid seems to be a good diagnostic option [64]. The severity of EOM depends on IgE concentration and the antigen-specific IgE of fungus in the middle ear effusion. Apart from ANCA positivity, there is a higher eosinophil fraction encountered in EGPA than in EOM, but both show greater eosinophil counts than normal. The study shows that EGPA should be suspected in patients with eosinophilia of more than 33% [65]. Regardless of ANCA vasculitis type, even localized OMAAV showed higher levels of the MPO-DNA complex compared with the patients with EOM, in spite of the serum ANCA status at the time of sampling [64].

#### 6.1.1. Eosinophilic Otitis Media Diagnostic Criteria [66]

- Major: otitis media with effusion or chronic otitis media with eosinophil—dominant effusion.
- Minor:
  1. Highly viscous middle ear effusion;
  2. Resistance to conventional treatment for otitis media;
  3. Association with bronchial asthma;
  4. Association with nasal polyposis.

Definitive case: positive for major + two or more minor criteria.

Exclusion criteria: Churg–Strauss syndrome, hypereosinophilic syndrome.

### 6.2. IgG4-RD vs. OMAAV

Immunoglobulin G4-related disease (IgG4-RD) is a systemic condition characterized by an elevated serum IgG4 level, tumefaction, the tissue infiltration of IgG4-positive plasma cells, and fibrosis. Diagnostic criteria for IgG4-RD were proposed in 2011. It is important to remember this entity during the differentiation of ANCA vasculitis [67,68]. Histology may be helpful in diagnosing as it shows lymphoplasmacytic infiltration, storiform-type fibrosis, and increased numbers of IgG4-positive plasma cells, but there is one case of ANCA concomitant IgG4-RD described [68]. Moreover, there is the possibility of IgG4/multifocal fibrosclerosis (MFS)-related HP with certain diagnostic criteria established [50], making it more important to differentiate from AAV.

## 7. Treatment

At the beginning, it is important to start with a comparison of systemic and local treatment intensity. It is suggested that immune activity in patients with AAV localized to the ear is equivalent to activity in patients with systemic AAV, based on sIL2-R, CRP, and ANCA status. Therefore, we may need a treatment for OMAAV of equal intensity to that used for systemic AAV. As the hearing level upon diagnosis was worse in patients with AAV localized to the ear than in patients with systemic AAV, an earlier diagnosis might be needed to improve hearing outcomes [69].

### 7.1. Controlling the Treatment

There are no official clinical practice guidelines for the treatment of OMAAV. As a form of AAV, OMAAV should be treated with immunotherapy according to recommendations presented by EULAR. The different forms of treatment are proposed for localized and systemic manifestations. To control and evaluate therapy, BVAS and VDI questionnaires should be used. However, in most research, remission is defined as the absence of manifestations attributable to AAV for 3 months. Relapse is defined as the occurrence of new involvements or as the recurrence or worsening of a manifestation of clinical vasculitis following remission [30]. In OMAAV, it seems to be more important to control the possibility of recurrence by checking hearing outcomes and otoscopic evaluation—like the previously mentioned SCOT system—than ANCA titers measurements. The dilatation of vessels and redness of the tympanic membrane reflect the effects of treatment relatively early and sensitively. Otologic findings and hearing levels should be checked at least every 3 months [51].

Poor prognostic factors for unfavorable hearing and life outcomes in patients with OMAAV include the following [29,30]:

- Facial palsy;
- Hypertrophic pachymeningitis;
- Negativity for both MPO-ANCA and PR3-ANCA;
- Disease relapse;
- Treatment with GCs alone.

### 7.2. OMAAV Antibacterial Treatment

The antimicrobial treatment in OMAAV depends on the auricular manifestation of the disease. Firstly, taking swabs from ear discharge when it is present is the most important action, before antimicrobial agent usage. Studies show that there might be a predilection to specific bacteria depending on the type of middle ear inflammation. In OME, there is no discharge and there is an indication for watchful waiting for about 3 months of ANCA treatment. If there is no inflammatory exacerbation of the disease and remission of the vasculitis is achieved but the patient still has signs and symptoms of OME (conductive hearing loss, type B tympanometry, retraction, and immobilization of tympanic membrane), myringotomy and/or tympanostomy tube insertion can be performed. During this procedure, a swab should be taken. It is said that molecular tests should be conducted, as they are much more sensitive and specific than traditional cultures [70,71]. Studies show that the most common pathogens in OME (either acute or chronic) are Haemophilus influenzae followed by Streptococcus pneumoniae and Moraxella cataralis. Alternatively, swabs from the nasopharynx (pharyngeal tonsil) can be taken instead of the middle ear, as the pathogens in the pharyngeal biofilm match the ones in the middle ear. In the case of acute otitis media or the exacerbation of OME, amoxicillin with a beta-lactamase inhibitor (45/mg/kg/day divided into two or three doses or 90mg/kg/day if the resistant pneumococci are suspected) should be prescribed. For patients allergic to penicillin, second- or third-generation cephalosporins, clindamycin, or macrolides should be used. Azithromycin and cefaclor are not advised due to their high resistance indices. If there is acute inflammation without perforation and discharge, myringotomy with a swab should be performed. Also, patients should be addressed by the attending physician, as it could be the beginning of vasculitis relapse. In chronic suppurative otitis media (CSOM) and chronic otitis media (COM) with cholesteatoma, antimicrobial agents should be started only if there is an exacerbation. The most frequently isolated are *Pseudomonas aeruginosa*, Staphylococcus aureus, and coagulase-negative staphylococci followed by Enterobacteriaceae—mainly Escherichia coli, Klebsiella pneumoniae, and Proteus species [70,72]. There are also anaerobic bacteria species such as Peptostreptococcus sp., Prevotella, Porphyromonas, Bacteroides, and Fusobacterium. Empiric treatment should cover both aerobic and anaerobic pathogens. The usage of topical agents and ear toilet in combination with systemic drugs is the best option in cases with ear drum perforation and discharge [70,72,73]. The most common topical drugs are drops with ciprofloxacin, ceftizoxime, aminoglycosides, polymyxin B combination ±gramicidin, trimethoprim/sulphacetamide/polymixin B combination, rifampicin, and chloramphenicol. Although the most common are quinolones and aminoglycosides, there is uncertainty about the relative effectiveness of different types of antibiotics [74].

### 7.3. Pharmacology

OMAV is part of a systemic disease called ANCA-associated vasculitis and, as a component of AAV, needs systemic immunosuppressive therapy. The type of immunosuppression depends on the type and the severity of the disease. Initially, the range of organ involvement should be established, as well as the diagnosis of whether the course of the disease is organ-/life-threatening or not. The therapeutic schedule of AAV therapy consists of two phases of the treatment. The first one is an induction therapy which lasts about 6 months and the second one is called maintenance therapy, which lasts 24–48 months. The latest EULAR recommendations for AAV treatment were established in 2022 and published in 2023 [75].

**Figure 1:**
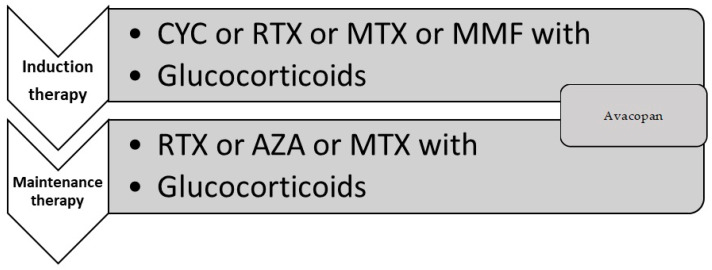
Therapeutic schedule for granulomatosis with polyangiitis (GPA) or microscopic polyangiitis (MPA) according to EULAR recommendations. AZA—azathioprine; CYC—cyclophosphamide; GC—glucocorticoid; MMF—mycophenolate mofetil; MTX—methotrexate; RTX—rituximab.

For granulomatosis with polyangiitis (GPA) or microscopic polyangiitis (MPA) with an organ- or life-threatening course of the disease, rituximab (RTX) or cyclophosphamide (CYC) with concomitant glucocorticoids (GC) and/or avacopane are recommended. The choice between RTX and CYC should be based on the medical data such as the clinical course of the disease, patient’s comorbidities or contraindication to one of the drugs, and procreation plans. RTX is preferred over CYC in cases of relapsing disease, a significant cumulative dose of CYC previously received, or young individuals with childbearing potential. The therapeutic schedule for GPA or MPA is summarized in Figure 1. Two schedules of RTX therapy are available. Both of them have similar efficacy and safety profiles, which were presented in the meta-analysis [76]. The first of them, approved in the European Union, assumes four infusions of 375 mg/m^2^ in one-week intervals [75]. The second one consists of two doses of 1g infusions in weeks 0 and 2 according to the schedule approved for rheumatoid arthritis [75]. The intravenous infusion of RTX should be preceded with a premedication protocol, including paracetamol (acetaminophen), clemastine, and GC to prevent cytokine release syndrome. Cyclophosphamide therapy is used mostly in the form of intravenous infusions in doses of 15 mg/kg in 2–3-week intervals according to the CYCLOPS study protocol [77]. The dose of CYC should be reduced in the case of kidney failure or/in elderly patients. The oral CYC daily dose is 2 mg/kg; however, the drug is currently rarely used because of the risk of high cumulative dose and its serious side effects [77].

The recommendations for the pharmacology treatment of AAV formed by various societies can be slightly different. However, these differences mostly concern the preferences between CYC and RTX in the induction therapy of organ- or life-threatening courses of AAV. The Japan Otological Society published in 2021 slightly emphasizes CYC usage, especially in the case of renal involvement, but in the update published in 2024, both drugs are recommended alternatively [29,78]. This update also introduces avacopane as an alternative to high doses of GC. Recommendations of the American College of Rheumatology (ACR) prefer the usage of RTX over CYC as a first-line induction therapy [79].

Non-organ-/life-threatening forms of the disease can be treated with rituximab, methotrexate (15–25 mg/week), or mycophenolate mofetil (2 g/day) with concomitant glucocorticoids and/or avacopan. The quick tapering of daily GC doses to reach the dose of 5 mg per day after 4–5 months is recommended [75].

The new drug, avacopan, blocks the C5a receptor on neutrophils. C5a is a terminal molecule produced during complement activation through the alternative pathway. As a result, avacopan inhibits neutrophil activation and chemoattraction, which play a crucial role in the pathogenesis of AAV [76]. Avacopan, in induction therapy, added to the short course of GC treatment (21 weeks) and had a similar remission rate at 26 weeks in comparison to patients treated with standard GC doses. However, in patients with glomerulonephritis, avacopan was better in terms of the recovery of renal function [76].

After 6 months, an evaluation of the induction therapy is necessary. If the remission of AAV is diagnosed, maintenance therapy should be introduced. Apart from a low dose of GCs (prednisone 5mg/day), the following drugs are used in maintenance therapy: rituximab, azathioprine (AZA), or methotrexate (MTX). Up to now, avacopan usage has been limited to 1 year of treatment and is not approved in many countries. According to the MAINRITSAN study, the most efficient drug in maintenance therapy is rituximab, with a relapse rate of 5% in comparison to 29% for azathioprine [77]. The therapeutic mode for maintenance therapy based on this trial is composed of five intravenous infusions of RTX in doses of 500 mg in the following schedule: 0–2 weeks and then at 6, 12, and 18 months. The recommended daily dose of AZA is 2 mg/kg. The duration of the maintenance treatment should last 24–48 months. Most recommendations (ACR 2021, EULAR 2022, Japan Research Committee 2023) favor RTX usage as a maintenance therapy [71,75,78,79,80].

Eosinophilic granulomatosis with polyangiitis (EGPA) with organ-/life-threatening forms of the disease should also be treated with CYC or RTX with concomitant GC. Non- organ-/life-threatening forms of the disease should use GCs as a first-line therapy. In the case of a refractory relapsing course of the disease, mepolizumab with GCs should be recommended. Mepolizumab (MEPO) is a monoclonal antibody against the alpha component of the receptor for IL-5 located on the surface of eosinophils. The clinical trial MIRRA revealed that MEPO was more effective than standard therapy and a significant reduction in GC dosage was possible [81].

**Figure 2:**
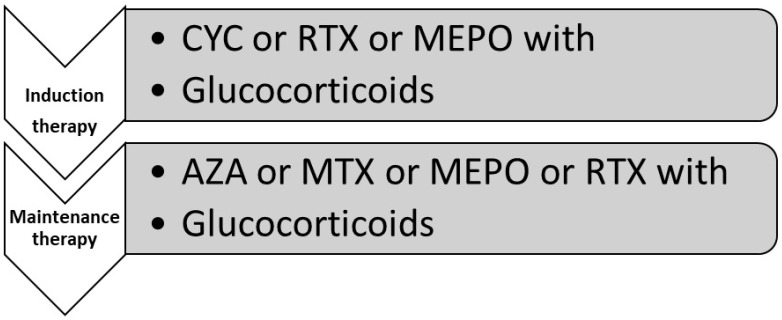
Therapeutic schedule for eosinophilic granulomatosis with polyangiitis (eGPA) according to EULAR recommendations. AZA—azathioprine; CYC—cyclophosphamide; GC—glucocorticoid; MEPO—mepolizumab; MTX—methotrexate; RTX—rituximab.

As a maintenance therapy for the disease, azathioprine, methotrexate, mepolizumab, or rituximab and GC in reduced doses can be used. The therapeutic schedule for eGPA is summarized in Figure 2.

### 7.4. Surgery

Firstly, during the active phase of vasculitis, ear surgeries such as tympanoplasty or mastoidectomy should not be performed, as they are not effective and may worsen the disease [25,29,45,82,83]. Some authors claim otherwise; myringotomy with tympanostomy tube placement is sometimes described to alleviate acute symptoms of middle ear inflammation and prevent the atelectasis of the tympanic membrane [40,84,85,86]. They are and preferably will be handy in taking swabs from middle ear fluid, not only for bacteriological or biochemical tests but also for tittering ETosis in the future. On the other hand, several case reports have shown that ear conditions worsen after myringotomy [87,88]. Another questionable thing is the local administration of steroids, where results are not unequivocal [27]. Maybe performing surgery during remission with steroids and immunosuppressants [84] in conductive or mixed hearing loss cases may be some kind of way to preserve hearing. In such cases, hearing impairment could be a manner of bad ventilation of the middle ear and not the vasculitis itself.

### 7.5. Cochlear Implantation

As it was said before, surgeries in OMAAV are definitely not the first line of treatment. The nationwide Japan survey documented hearing outcomes for treated cases of OMAAV followed for 24 months, where a complete/marked recovery (30%), partial recovery (30%), or nonrecovery (40%) were reported. Complete deafness was seen in 7.2% of cases, and complete bilateral deafness in 3.5% [29]. In other cases, there was progressive and irreversible hearing loss or even deafness upon the first visit. Moreover, these cases are also refractory to immunosuppressive therapy. Cochlear implant (CI) surgery is a treatment worth considering, but the results are not yet coherent. Indications for cochlear implantation for patients with OMAAV do not differ from the general population (Section 7.5.1) [89]. The protocol for device selection is also the same. Having no contraindications for surgery is significant, so performing surgery in the remission phase seems to be crucial. Some cases show unfavorable outcomes for this method in spite of good overall control of ANCA disease [40]. The reasons for the poor results of the therapy are probably the destruction of the blood–labyrinthine barrier and the rapid progression of sensorineural hearing loss. On the other hand, there are two cases where speech recognition after CI implantation ranges from 40 to 56% alone and from 76 to 96% with speech and oral forms. The beneficial effect of a CI depends on how well the spiral ganglion is preserved but there is no method to detect and measure it [40,80]. Another important factor is the amount of calcification and granulation in the cochlea, which may worsen results or even prevent surgery because of mechanical reasons. In such cases, ears with less calcification should be chosen for implantation. Also, using a depth gauge to widen the round window is recommended [84]. There is also a case of slowly progressing bilateral sensorineural hearing loss. A ventilatory tube was placed on the inferior ear accompanied by steroids and immunosuppressants prior to CI implantation. Because of the good response to treatment after 3 months, a subtotal petrosectomy was not performed. CI surgery with a facial recess approach was used with good outcomes—75% of monosyllabic word discrimination score vs. 0% before surgery and 25 dB of speech reception threshold vs. none. In patients who do not respond to pharmacological treatment, subtotal petrosectomy prior to CI surgery is indicated [85]. The possibility of using combined electroacoustic stimulation is also a good way to improve hearing outcomes in certain patients with residual hearing loss in the low frequencies [90].

#### 7.5.1. Current Adult Selection Criteria for Cochlear Implantation [89]

1. Severe or profound hearing loss with a pure-tone average (PTA) of 70 dB or greater hearing level (HL);
2. Use of appropriately fitted hearing aids or a trial with amplification;
3. Aided scores on open-set sentence tests of less than 50% in the ear to be implanted and 60% in the contralateral ear;
4. No evidence of central auditory lesions or lack of an auditory nerve;
5. No evidence of contraindications for surgery in general or CI surgery in particular.

## 8. Conclusions

All things considered, ANCA vasculitides are systemic diseases and require interdisciplinary collaboration. The goal of this publication is to sum up and underline the problem of the aural manifestation of AAV. It details the definition of OMAAV and allows for a better understanding of the specific tasks of medical professionals taking part in diagnosing and treating the disease. Among others, this publication is directed to otolaryngologists who may encounter patients with AAV with or without a diagnosis, describing how valid early diagnosis is. It gives a clue into how to manage patients with ANCA and clarifies cooperation with internal medicine doctors. Unfortunately, this paper shows how little we know about ANCA vasculitides and particularly OMAAV. It is our duty to discover the nature of this entity and raise awareness of it. Our center is currently conducting a study about otologic manifestations in patients with diagnosed ANCA vasculitides. Hopefully, our study may help to better comprehend the course of hearing loss in OMAAV and enable the early recognition of it, before aggravation.

## Data Availability

No new data were created or analyzed in this study. Data sharing is not applicable to this article.
